# Effects of Polishing and Artificial Aging on Mechanical Properties of Dental LT Clear^®^ Resin

**DOI:** 10.3390/jfb14060295

**Published:** 2023-05-25

**Authors:** Anna Paradowska-Stolarz, Joanna Wezgowiec, Andrzej Malysa, Mieszko Wieckiewicz

**Affiliations:** 1 Division of Dentofacial Anomalies, Department of Maxillofacial Orthopedics and Orthodontics, Wroclaw Medical University, 50-425 Wroclaw, Poland; 2 Department of Experimental Dentistry, Wroclaw Medical University, 50-425 Wroclaw, Poland; joanna.wezgowiec@umw.edu.pl (J.W.); andrzej.malysa@umw.edu.pl (A.M.)

**Keywords:** 3D print, resin, dental LT clear, polishing, artificial aging, compression, tensile modulus

## Abstract

Three-dimensional printing has become incorporated into various aspects of everyday life, including dentistry. Novel materials are being introduced rapidly. One such material is Dental LT Clear by Formlabs, a resin used for manufacturing occlusal splints, aligners, and orthodontic retainers. In this study, a total of 240 specimens, comprising two shapes (dumbbell and rectangular), were evaluated through compression and tensile tests. The compression tests revealed that the specimens were neither polished nor aged. However, after polishing, the compression modulus values decreased significantly. Specifically, the unpolished and nonaged specimens measured 0.87 ± 0.02, whereas the polished group measured 0.086 ± 0.03. The results were significantly affected by artificial aging. The polished group measured 0.73 ± 0.05, while the unpolished group measured 0.73 ± 0.03. In contrast, the tensile test proved that the specimens showed the highest resistance when the polishing was applied. The artificial aging influenced the tensile test and reduced the force needed to damage the specimens. The tensile modulus had the highest value when polishing was applied (3.00 ± 0.11). The conclusions drawn from these findings are as follows: 1. Polishing does not change the properties of the examined resin. 2. Artificial aging reduces resistance in both compression and tensile tests. 3. Polishing reduces the damage to the specimens in the aging process.

## 1. Introduction

Three-dimensional (3D) printing is becoming one of the most popular methods for fabricating customized dental elements in contemporary dentistry. It is widely employed for various applications, including dental restorations, personalized orthodontic and prosthetic appliances, as well as precise components such as surgical guides [1,2]. The ability to create precise and individualized elements has propelled 3D printing materials to the forefront of modern dentistry, enabling in-house treatment planning [3].

Clear aligners have emerged as a popular trend in modern orthodontics, affecting various branches of dentistry. While thermoformed plates have traditionally been used for their fabrication, there is a growing preference for 3D printed materials due to their enhanced accuracy and precision. The integration of digital technologies has prompted dentists and dental companies to explore novel materials, among which Dental LT Clear resin (Formlabs) stands out. This material is classified as a IIa biocompatible resin, making it suitable for long-term use on the skin and mucosal surfaces. In addition to its high translucency, Dental LT Clear resin exhibits nonlinear compression resistance of up to 600 N, comparable to the biting force. This value is comparable to other popular materials commonly used for fabricating clear aligners, such as Duran and Durasoft [4].

Biocompatibility is a critical feature of dental materials, particularly when they come into contact with tissues for extended periods. Technical tests are employed to assess the biocompatibility of materials and ensure their safety for human use [5]. According to the manufacturer [6], Dental LT Clear resin is a new-generation, biocompatible material specifically designed for long-term applications. It is composed of several chemical components, including 7,7,9-(or 7,9,9)-trimethyl-4,13-dioxo-3,14-dioxa-5,12-diazahexadecane-1,16-diyl bismethacrylate, 2-hydroxyethyl methacrylate, *a* reaction mass of Bis(1,2,2,6,6-pentamethyl-4-piperidyl) sebacate and methyl 1,2,2,6,6-pentamethyl-4-piperidyl sebacate, diphenyl(2,4,6-trimethylbenzoyl)phosphine oxide, acrylic acid, monoester with propane-1,2-diol, ethylene dimethacrylate, 2-hydroxyethyl acrylate, mequinol, 4-methoxyphenol, and hydroquinone monomethyl ether. The material possesses translucency, rigidity, and strength, making it an ideal choice for esthetically pleasing individual appliances, including dental aligners. Additionally, the resin is used for fabricating occlusal guards, splints, and orthodontic retainers. It is important to note that food and beverages can affect the properties of intraoral appliances, diminishing their esthetics and compromising their structural integrity [7,8]. The properties of materials are also influenced by intraoral conditions. Originally, CAD/CAM materials were used for dental restorations (temporary and permanent ones) and the purpose of the use is longitudinal—which means that the pieces of material stay in contact with the oral cavity for a long time. Consequently, the properties of the materials are examined in terms of color stability and durability [8,9]. Recently, more materials have been introduced, and therefore doctors and laboratories are able to prepare more customized pieces, such as individual face masks or individual appliances for cleft patients [10,11,12].

Dental LT Clear is a relatively new material that has not yet been fully investigated [3,13]. Therefore, the study we have designed is among the first of its kind in our opinion. Apart from its intended use in intraoral splints, Dental LT Clear may also have potential applications in the fabrication of precise individual and orthopedic appliance components, such as nasal–alveolar molding plates for patients with clefts [13]. A similar rigid material, BioMed Amber, produced by the same manufacturer (Formlabs), has shown greater resistance to compression but lower resistance to tensile forces. This makes BioMed Amber more suitable for the fabrication of mouth guards and occlusal splints. However, it should be noted that BioMed Amber is not designed for long-term use and should only remain in contact with the human body for a short period [6].

In another study [14], a comparison of three dental 3D printed materials revealed that Dental LT Clear exhibited the greatest stability following compression and tensile tests. Fractal dimension and texture analyses showed minimal changes in the material’s properties. However, bone index analysis of BioMed Amber indicated a decline in material quality because of the performed tests. It should be noted that according to the information provided by the manufacturer [6], Dental LT Clear material is not recommended for sterilization. Nonetheless, a recent study [15] revealed the benefits of sterilization in terms of reducing monomer elution, and autoclaving at 132 °C for 4 min improved the microhardness of the resin. Therefore, sterilization should be considered during the prefabrication of occlusal splints. 

The aim of this study was to examine the mechanical resistance properties of a selected 3D printable resin, specifically Dental LT Clear from Formlabs, with regard to the effects of specimen polishing and aging. To evaluate the material, we formulated the following hypotheses:

- There is no influence of polishing on the material’s durability.
- There is no influence of artificial aging on the material’s durability.
- There is no relation between application of polishing and artificial aging regarding the material’s durability.
- Polishing does not change the properties of the material in terms of artificial aging.

## 2. Materials and Methods

### 2.1. Materials

In this study, we examined the properties of Dental LT Clear, a 3D printable biocompatible resin provided by Formlabs, located in Milbury, OH, USA. The specimens were prepared using the Form 2 printer from Formlabs, specifically designed for this resin. The printer is a self-adjusting device, which automatically sets the print settings once the cartridge is inserted. The printing process was carried out using violet light (405 nm) with a power output of 250 mW. The print layer thickness was set to 100 microns, and the temperature was maintained at 35 °C. The manufacturer’s recommendations regarding potential applications and properties of Dental LT Clear are summarized in Table 1.

### 2.2. Specimens’ Preparation and Artificial Aging

For this research, two types of specimens were prepared. The rectangular specimens were designed for the compression test following the ISO 604:2003 standard [16]. The dumbbell-shaped specimens (type 1BA) were prepared for the tensile test according to the ISO 527-1:2019(E) standard [17]. While the standards required a minimum of five samples, the authors decided to expand the test to 30 specimens for each test. In total, 240 specimens of Dental LT Clear resin were printed using the Form 2 printer by Formlabs, following the ISO standards and the manufacturer’s instructions. Of these, 120 were rectangular-shaped and 120 were dumbbell-shaped.

After printing, the specimens were rinsed twice for 10 min each in 99% isopropanol alcohol (Stanlab, Lublin, Poland). Following a 30 min drying period at room temperature, the specimens were postcured using Form Cure from Formlabs at 80 °C for 20 min, as recommended by the manufacturer for Dental LT Clear resin. Once the specimens were prepared, the supports were removed. All specimens were then ground using sandpaper, but only half of them (60 rectangular and 60 dumbbell-shaped) underwent further polishing on one side using 0.2 pumice (Everall 7, Warsaw, Poland) and polishing paste (Everall 7) with the Reiter Poliret Mini Feinwerktechnik (GmbH, Bad Essen, Germany). The polishing process involved a rotational range of 1000–4500 rotations per minute and an average speed of 2250 rpm.

Following preparation, the specimens were stored at room temperature and 50% humidity for 24 h (for the tensile test) or 4 days (for the compression test). Half of the specimens were tested immediately after the storage period, while the other half (60 of each shape) underwent artificial aging for 90 days in distilled water at 37 °C. The water was changed weekly, based on the scheme used in a previous study on conventional dental restorative materials [18]. The decision to change the water every 7 days was made to prevent any potential alteration of properties while ensuring that the water did not evaporate.

### 2.3. Compression Test

According to the ISO 604:2003 [15] standard, specimens measuring (10.0 ± 0.2) mm × (10.0 ± 0.2) mm × (4 ± 0.2) mm were selected for testing. Prior to the test, the specimens were conditioned for 4 days at 23 °C/50% relative humidity (RH) in ambient air. The height and width of the specimens were then measured at five points using a Magnusson digital caliper (150 mm) (Limit, Wroclaw, Poland). The mean values of these measurements were calculated.

**Figure 1:**
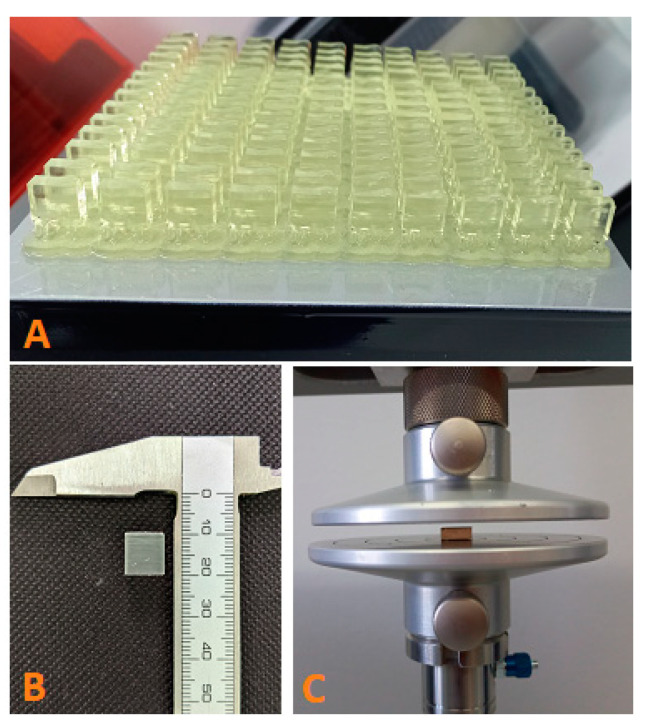
Compression test: (**A**) set of specimens after printing; (**B**) a finished specimen before compression; (**C**) resin specimen between the compression plates.

Axial compression tests were conducted using the Z10-X700 universal testing machine from AML Instruments in Lincoln, UK. The tests were performed at a constant speed of 1 mm/min (Figure 1). By recording the uniaxial stress–strain curve, the compressive modulus (E [MPa]) of each specimen was determined using the slope of the curve. The changes in width and height during the compression test were compared to the measurements taken before and after compression, as shown in Table 2.

### 2.4. Tensile Test

The dumbbell-shaped specimens (type 1BA) were 3D printed with a length of 75 mm and an end width of 10 mm, while the thickness was 2 mm. These measurements adhered to the ISO 527-2:2019 standard [17]. Prior to the tensile test, the specimens were conditioned at room temperature (23 °C) and 50% RH for 24 h. Using a Magnusson digital caliper (150 mm) (Limit, Wroclaw, Poland), the width and height of the specimens were measured at the test length, with measurements taken at five points. The mean values of these measurements were then calculated.

**Figure 2:**
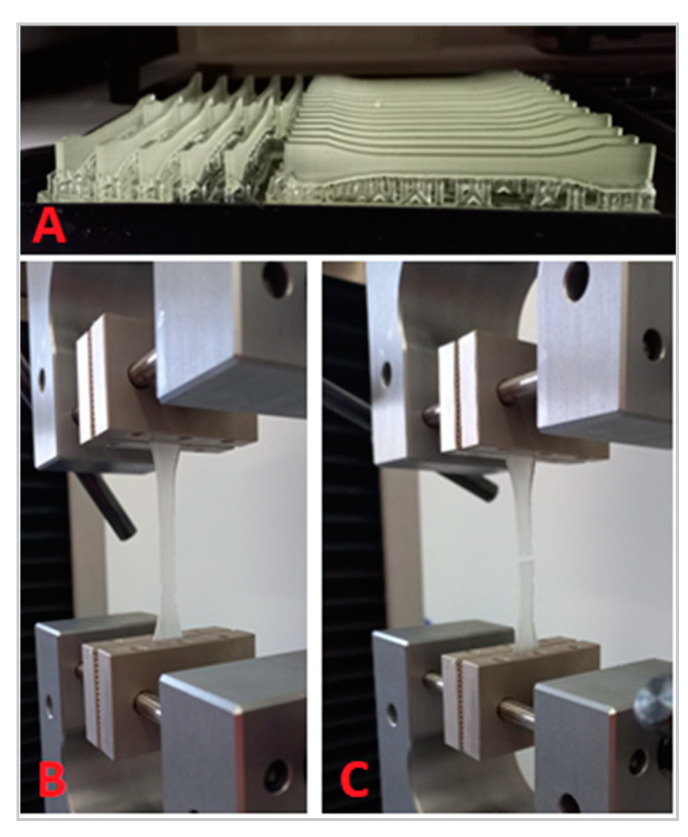
Tensile test: (**A**) set of specimens after printing; (**B**) a finished specimen mounted between grips before tensile force application; (**C**) a specimen broken by tensile force.

To perform the tensile test, a universal testing machine (Z10-X700, AML Instruments, Lincoln, UK) was utilized. The test was conducted at a constant speed of 5 mm/min, as shown in Figure 2. If any of the specimens broke outside of the test length, they were discarded. Based on the measurements obtained during the test, the stress and strain of the specimens were determined. The formulas for these calculations are presented in Table 2.

### 2.5. Statistical Analysis

The statistical analysis was conducted using Statistica c. 13 software (TIBCO Software Inc., Palo Alto, CA, USA).

The analysis involved calculating the mean values, along with their corresponding standard deviations, for both the compression and tensile modulus of the specimens. To assess any potential statistical differences between the specimens, the Kruskal–Wallis test by rank was employed, with a *p*-value threshold set at the range of *p* < 0.001. To compare the results obtained from the four tests for each trial, a multivariate analysis of variance (MANOVA) test was conducted. Finally, to determine the significance of the presented results, the Mann–Whitney U test was performed.

## 3. Results

The results of the conducted tests are summarized and presented in three tables and four figures. Table 3 provides an overview of the elasticity modulus measurements of Dental LT Clear resin. It was found that the highest compression modulus values were observed in specimens that had not undergone polishing or aging. On the other hand, specimens subjected to aging or both polishing and aging exhibited lower mean compression modulus values. It is worth noting that the tensile test results showed the widest ranges when polishing was applied without aging, while the narrowest ranges were observed after the application of aging. 

**Figure 3:**
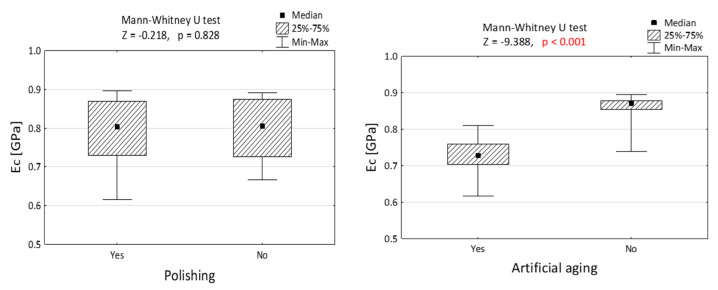
Module of elasticity in compression (E_c_) of Dental LT Clear resins, differing in polishing and artificial aging. The Mann–Whitney U test was performed to validate the importance of the result. *p* value lower than 0.001 is presented in red.

**Figure 4:**
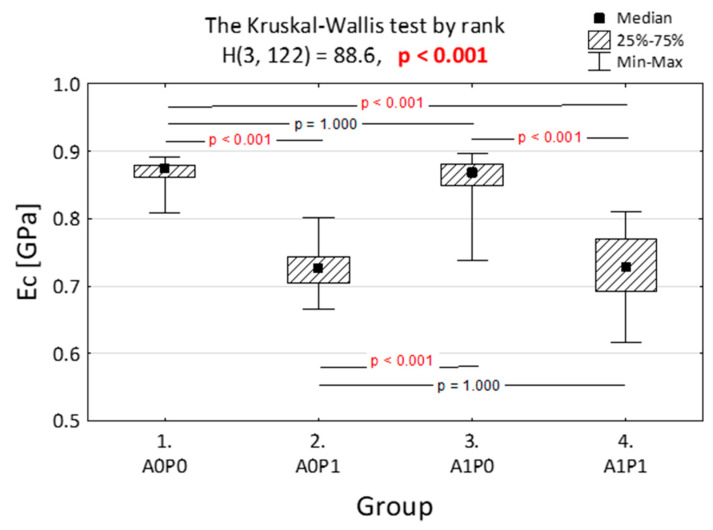
Module of elasticity in compression (E_c_) of the Dental LT Clear specimens after different technological treatments (A0P0—no artificial aging and polishing, A0P1—no artificial aging, after polishing, A1P0—artificial aging applied without polishing, and A1P1—both polishing and artificial aging). The Kruskal–Wallis and post hoc tests were applied. *p* value lower than 0.001 is presented in red.

Figure 3 and Figure 4 depict the comparisons of the elasticity modulus. Figure 3 illustrates that the impact of polishing on compression resistance is minimal, while artificial aging noticeably weakens the material properties by reducing the force required to damage the specimens. This difference is statistically significant (*p* < 0.001). In Figure 4, the largest disparity is observed when comparing the nonpolished and nonaged group with the group of specimens that underwent both polishing and aging.

Figure 4 displays the variance analysis conducted to determine the influence of artificial aging and polishing on the module of elasticity in compression (*E*_c_). The mean values of the elastic modulus varied depending on whether artificial aging was applied with or without polishing. Polishing had an impact on the nonaged specimens, but when comparing the aged specimens, polishing itself did not influence the material’s properties. 

Additionally, Table 4 shows the results of the MANOVA test, indicating that there were statistically significant differences among the results obtained from all the processes performed on the specimens. 

In contrast to the previous results, Table 5 presents the MANOVA test results for the module of elasticity in applied tension. It shows that the only significant influence observed was due to artificial aging. There was no observed influence of the interaction between polishing and artificial aging. 

**Figure 5:**
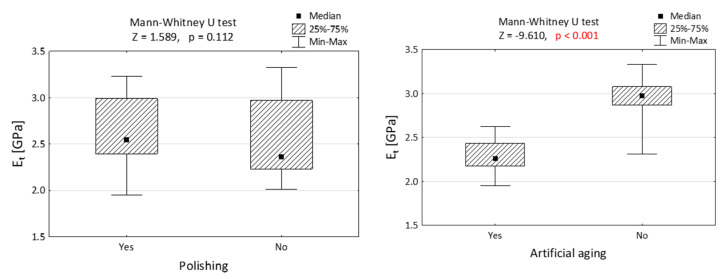
Module of elasticity in tension (E_t_) of specimens made of Dental LT Clear resin, differing with polishing and artificial aging. Results presented in the form of Mann–Whitney U test. *p* value lower than 0.001 is presented in red.

The results depicted in Figure 5 demonstrate that polishing had no significant influence on the tensile properties of the examined resin. However, artificial aging was found to reduce the resistance to breakage during the tensile test.

**Figure 6:**
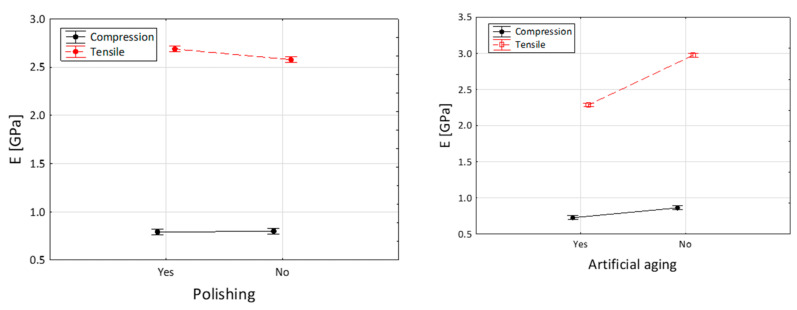
The influence of polishing and artificial aging on the compression and tensile modulus of Dental LT Clear.

Figure 6 provides a summary of the previous findings, indicating that polishing has a slight positive effect on the resistance to tension, but it does not affect the resistance to compression. In contrast, artificial aging decreases the force required to damage the specimens in both the compression and tensile tests, although the impact is less noticeable in compression. Additionally, the tensile modulus value significantly decreases with the application of artificial aging.

## 4. Discussion

Dental LT Clear, as a class IIa biocompatible resin, is specifically designed for long-term use and contact with tissues, including oral mucosa. It is commonly utilized in the production of clear aligners and dental guards, which are intended for extended wear by patients [4,19]. These splints are designed for long-term use and stay in the patient’s mouth for several hours per day and should not be used while eating and drinking due to the potential loss of color stability and mechanical properties [7,20]. With this in mind, we conducted this study to evaluate the mechanical properties of Dental LT Clear resin when subjected to two technological activities: polishing and artificial aging.

Considering that the examined material is designed for long-term use, such as in splints, aligners, and retainers [6], we decided to assess its resistance to artificial aging to simulate this condition. We chose to use water for artificial aging to mimic the humid environment of the oral cavity. Thermocycling, which involves temperature variations during eating, was not considered in our study [19]. Water storage provides valuable information on hydrolytic degradation [21]. Patient appliances should be polished to create smoother surfaces and prevent irritations. The results we obtained supported the notion that polishing helps protect the resin from unfavorable conditions, thereby confirming the durability of its mechanical properties. However, it is important to note that Dental LT Clear is a transparent, clear resin and is not intended for dental fillings. Therefore, thermocycling was not included as a method of material aging. We found a study [22] that examined the influence of artificial aging on the material’s properties. Although their study had a similar design to ours, they assessed the resistance to mechanical forces after 2 and 4 weeks. The two-week period, presented by Reymus and Stawarczyk [22], provides insights into the material’s durability within a clear aligner treatment timeframe. In contrast, our study was conducted over a period of 3 months, which is a more appropriate timeframe for assessing the long-term use of Dental LT Clear as an occlusal splint or retainer. It is also worth mentioning that other resins, such as Tera Harz TC-85 (Graphy), have been studied in more detail in the context of artificial aging [3]. Dental LT Clear is a relatively new resin and, therefore, the available studies on this specific topic are limited. 

Dental LT Clear is indeed a relatively new material, and during our literature search, we did not come across articles specifically examining the features presented in this research. Existing studies on resin use in dentistry often focus on color changes, which is a primary concern for researchers [23,24,25,26]. Our study demonstrates that polishing has minimal impact on the mechanical resistance of Dental LT Clear, while artificial aging significantly weakens these properties. Therefore, we believe that our paper holds value and importance, especially for clinicians. Over time, splints may become less precise and show signs of wear. Another interesting study revealed that printed splints tend to be thicker than the designed file, suggesting potential loss of precision during use [27]. Scanning, which replaces traditional intraoral impressions, can also introduce errors and distortions [28]. Additionally, the properties of impressions may change during disinfection. Silicone materials are known to be more resistant to disinfecting agents and sterilization, whereas commonly used alginate materials are less stable and accurate, losing their precision after undergoing antibacterial procedures [29,30,31].

An interesting observation is that the angle of printing is crucial in the context of polishing, as the layers of material used for occlusal splint preparation can result in irregularities and reduced precision in the structure of the splint [32]. However, this aspect was not the focus of our study, as we did not plan to print the specimens at different angles. 

In a comparative study, it was found that Dental LT Clear has the highest fracture rate among other resins used for occlusal splints [33]. This finding highlights the importance of considering the properties of different materials when planning any type of splint. It also emphasizes the need for further research and the development of new materials with more stable properties for similar applications.

Furthermore, a study by Paradowska-Stolarz et al. [14] demonstrated that the application of external forces does not significantly alter the fractal dimension and texture analysis reveals only resistance to compression—the study shows that Dental LT Clear remains stable in its mechanical features, which indicates that the microscopic structure of the material remains relatively unchanged after undergoing the tested conditions. The study also revealed the high stability of Dental LT Clear against mechanical action, as indicated by its resistance to compression.

It is worth mentioning that 3D composite materials may absorb water and undergo changes in weight. Although this feature was not evaluated in our study, it is an interesting finding that warrants further investigation [34].

We acknowledge that our research has certain limitations. One of the main limitations is that we only focused on one resin, Dental LT Clear, without comparing it to other materials. However, we believe that our study is a novel contribution to this field and was designed with this specific material in mind. It is worth noting that other papers in the literature tend to concentrate on restorative and prosthetic materials [23,24,25,26], whereas our study specifically examines a material used for occlusal splints and clear aligners.

ISO standards typically suggest using five samples for this type of research. However, by expanding our sample size to 30 specimens, we believe that our study gains a significant advantage in terms of statistical analysis and the reliability of our findings. Furthermore, the scarcity of references on this particular topic underscores the novelty and originality of our research.

## 5. Conclusions

Based on the obtained results, we can draw several conclusions. Firstly, polishing has minimal influence on the properties of Dental LT Clear resin. However, artificial aging significantly affects both the compressive modulus and tension of the material. Secondly, polishing increases the resistance of the specimens to artificial aging, as evidenced by the higher force required to damage the specimens. Therefore, it is recommended to polish appliances made from Dental LT Clear resin after printing to enhance their durability and resistance to wear during use.

## Figures and Tables

**Table 1:** A brief description of Dental LT Clear applications recommended by the producer.

Resin	Application
Dental LT Clear Resin	Characteristics: Long-term use Biocompatible Suitable for mucosal and skin contact Highly esthetic Transparent, translucent Strong, rigid Use: Hard splints Occlusal guards Retainers Aligners Other direct-printed long-term orthodon-tic appliances

**Table 2:** The formulas for calculation of the compression and tensile modulus.

	Formula	Explanations
Compressive modulus	Compressive stress $\sigma =\frac{F}{A}\left[\mathrm{MPa}\right]$ Nominal strain $\varepsilon =\frac{\Delta L}{L}$	F—force [N] A—initial cross sectional area measurement [mm^2^] L—the initial distance between the compression plates [mm] ΔL—the decrease in the distance between the plates after the test [mm]
Tensile modulus	Tensile stress $\sigma =\frac{F}{A}\left[\mathrm{MPa}\right]$ Nominal strain $\varepsilon =\frac{\Delta L}{L}$ $Et=\frac{\sigma_{2}-\sigma_{1}}{\varepsilon_{2}-\varepsilon_{1}}\left[\mathrm{MPa}\right]$	F—force [N] A—initial cross sectional area measurement [mm^2^] L—the initial distance between the grips [mm] ΔL—the increase in the distance between the grips after the test [mm] *σ*1—*the stress in MPa* measured at a strain of 0.0005 (*ε*1) *σ*2—*the stress in MPa* measured at a strain of 0.0025 (*ε*2)

**Table 3:** The measurements of elasticity module of Dental LT Clear resin after the compression (E_c_) and tensile (E_t_) tests.

E (GPa)	Polishing	Aging	N	M ± SD	Me [Q1–Q3]	Min–Max
Compression E_c_	No	No	30	0.87 ± 0.02	0.87 [0.86–0.88]	0.81–0.89
	No	Yes	30	0.73 ± 0.03	0.73 [0.71–0.74]	0.67–0.80
	Yes	No	32	0.86 ± 0.03	0.87 [0.85–0.88]	0.74–0.90
	Yes	Yes	30	0.73 ± 0.05	0.73 [0.69–0.77]	0.62–0.81
Tensile E_t_	No	No	32	2.96 ± 0.20	2.97 [2.84–3.07]	2.31–3.33
	No	Yes	32	2.20 ± 0.10	2.23 [2.13–2.26]	2.01–2.39
	Yes	No	28	3.00 ± 0.11	3.00 [2.93–3.08]	2.77–3.23
	Yes	Yes	36	2.38 ± 0.16	2.43 [2.25–2.51]	1.95–2.62

M—mean, SD—standard deviation, Me—median (50th percentile), Q1—lower quartile (25th percentile), Q3—upper quartile (75th percentile), Min—smallest value, Max—greatest value.

**Table 4:** MANOVA test for elasticity module for Dental LT Clear resin in compression test. *p* value lower than 0.001 is presented in red.

Effect	SS	df	MS	*F*	*p*
Constant	731.1	1	731.1	60901	<0.001
Direct	210.1	1	210.1	17502	<0.001
Polishing	0.166	1	0.166	13.8	<0.001
Artificial aging	10.60	1	10.60	883	<0.001
Direct Polishing	0.213	1	0.213	17.7	<0.001
Polishing + Artificial aging	4.773	1	4.773	398	<0.001
Artificial aging + Polishing	0.078	1	0.078	6.49	0.011
Direct Polishing + Artificial aging	0.060	1	0.060	5.02	0.026
Error	2.9052	242	0.012		

**Table 5:** MANOVA test for module of elasticity in tension of Dental LT Clear resin. *p* value lower than 0.001 is presented in red.

Effect	SS	df	MS	*F*	*p*
Constant	77.10	1	77.10	62136	0.000
Polishing	0.001	1	0.001	1.16	0.283
Artificial aging	0.562	1	0.562	452.6	0.000
Artificial aging + Polishing	0.001	1	0.001	0.44	0.506
Error	0.146	118	0.001		

## Data Availability

The data presented in this study are available on request from the corresponding author. The data are not publicly available due to privacy.
